# Predicting Episodes of Hypovigilance in Intensive Care Units Using Routine Physiological Parameters and Artificial Intelligence: Derivation Study

**DOI:** 10.2196/60885

**Published:** 2025-08-27

**Authors:** Raphaëlle Giguère, Victor Niaussat, Monia Noël-Hunter, William Witteman, Tanya S Paul, Alexandre Marois, Philippe Després, Simon Duchesne, Patrick M Archambault

**Affiliations:** 1 Department of Computer Sciences, Faculty of Sciences and Engineering, Université Laval, Québec, QC, Canada; 2 Centre de recherche intégrée pour un système apprenant en santé et services sociaux, Centre intégré de santé et de services sociaux de Chaudière-Appalaches, Lévis, QC, Canada; 3 Thales Research and Technology Canada (TRT-CA), Québec, QC, Canada; 4 Mathematics and Computer Science Department, École Centrale de Lille, Lille, France; 5 School of Psychology, Université Laval, Québec, QC, Canada; 6 Québec Heart and Lung Institute, Université Laval, Québec, QC, Canada; 7 Department of Physics, Engineering Physics and Optics, Faculty of Sciences and Engineering, Université Laval, Québec, QC, Canada; 8 Department of Radiology and Nuclear Medicine, Faculty of Medicine, Université Laval, Québec, QC, Canada; 9 Department of Family Medicine and Emergency Medicine, Faculty of Medicine, Université Laval, Ferdinand Vandry Pavillon, 1050 Av. de la Médecine, Québec, QC, G1V 0A6, Canada, 1 418-656-5245; 10 VITAM - Centre de recherche en santé durable, Québec, QC, Canada; 11 Department of Anesthesiology and Intensive Care, Faculty of Medicine, Université Laval, Québec, QC, Canada

**Keywords:** vigilance, hypovigilance, hypoactive delirium, machine learning, detection model, physiological parameters, automated monitoring, intensive care unit, ICU, delirium, hyperactive, monitoring, detection, prediction model, artificial intelligence

## Abstract

**Background:**

Delirium is prevalent in intensive care units (ICUs), often leading to adverse outcomes. Hypoactive delirium is particularly difficult to detect. Despite the development of new tools, the timely identification of hypoactive delirium remains clinically challenging due to its dynamic nature, lack of human resources, lack of reliable monitoring tools, and subtle clinical signs including hypovigilance. Machine learning models could support the identification of hypoactive delirium episodes by better detecting episodes of hypovigilance.

**Objective:**

Develop an artificial intelligence prediction model capable of detecting hypovigilance events using routinely collected physiological data in the ICU.

**Methods:**

This derivation study was conducted using data from a prospective observational cohort of eligible patients admitted to the ICU in Lévis, Québec, Canada. We included patients admitted to the ICU between October 2021 and June 2022 who were aged ≥18 years and had an anticipated ICU stay of ≥48 hours. ICU nurses identified hypovigilant states every hour using the Richmond Agitation and Sedation Scale (RASS) or the Ramsay Sedation Scale (RSS). Routine vital signs (heart rate, respiratory rate, blood pressure, and oxygen saturation), as well as other physiological and clinical variables (premature ventricular contractions, intubation, use of sedative medication, and temperature), were automatically collected and stored using a CARESCAPE Gateway (General Electric) or manually collected (for sociodemographic characteristics and medication) through chart review. Time series were generated around hypovigilance episodes for analysis. Random Forest, XGBoost, and Light Gradient Boosting Machine classifiers were then used to detect hypovigilant episodes based on time series analysis. Hyperparameter optimization was performed using a random search in a 10-fold group-based cross-validation setup. To interpret the predictions of the best-performing models, we conducted a Shapley Additive Explanations (SHAP) analysis. We report the results of this study using the TRIPOD+AI (Transparent Reporting of a multivariable prediction model for Individual Prognosis Or Diagnosis for machine learning models) guidelines, and potential biases were assessed using PROBAST (Prediction model Risk Of Bias ASsessment Tool).

**Results:**

Out of 136 potentially eligible participants, data from 30 patients (mean age 69 y, 63% male) were collected for analysis. Among all participants, 30% were admitted to the ICU for surgical reasons. Following data preprocessing, the study included 1493 hypovigilance episodes and 764 nonhypovigilant episodes. Among the 3 models evaluated, Light Gradient Boosting Machine demonstrated the best performance. It achieved an average accuracy of 68% to detect hypovigilant episodes, with a precision of 76%, a recall of 74%, an area under the curve (AUC) of 60%, and an *F*_1_-score of 69%. SHAP analysis revealed that intubation status, respiratory rate, and noninvasive systolic blood pressure were the primary drivers of the model's predictions.

**Conclusions:**

All classifiers produced precision and recall values that show potential for further development, with slightly different yet comparable performances in classifying hypovigilant episodes. Machine learning algorithms designed to detect hypovigilance have the potential to support early detection of hypoactive delirium in patients in the ICU.

## Introduction

Delirium is defined by the *Diagnostic and Statistical Manual of Mental Disorders, 5th edition* (*DSM-5*) as a “transient disturbance of attention and awareness, manifested as a reduced ability to control, focus, maintain, and transfer attention and as a weakened orientation to the environment” [1]. As reported by Fiest et al [2], a missed or delayed diagnosis of delirium is associated with adverse outcomes, particularly in intensive care units (ICUs), where it can lead to prolonged hospital stays, increased mortality rates, slower recovery, and persistent cognitive impairment [2-4].

There are two types of delirium: hyperactive delirium, characterized by restlessness and agitation, and hypoactive delirium, which presents with low vigilance and apathy [5]. Hypoactive delirium is the most common form of delirium. It is often unrecognized due to the challenges of diagnosing this specific subtype of delirium [6]. Kiely [7] suggests that patients with hypoactive delirium may have a higher risk of mortality compared to other delirium subtypes. Despite its clinical relevance, hypoactive delirium is often undetected in routine clinical practice [6,8]. Improvements in screening and therapy have occurred, but the identification of hypoactive delirium still poses a serious challenge, given that the onset of episodes remains difficult to determine and fluctuates over time [9]. Furthermore, its detection is labor-intensive, requiring frequent reevaluation and clinical interpretation using bedside instruments and questionnaires [10]. These instruments and questionnaires become even harder to use when patients and health care providers speak different languages [11]. A systematic review of ICU delirium prediction models by Ruppert et al [3] also found that while many models performed well, they only predicted the condition using baseline admission data from a single point in time, not considering the dynamic nature of delirium.

The main symptom of hypoactive delirium is decreased vigilance, also known as hypovigilance [5]. As defined by van Schie et al [12], “vigilance is the ability to remain aware of relevant and unpredictable changes in an individual’s surrounding environment, regardless of whether such changes actually occur.” Van Schie also described delirium as two-dimensional. First, the level of alertness required to be vigilant, and second, the extent to which vigilance may increase or decrease over time [12].

Dynamic changes in a patient’s vigilance level can potentially be detected using continuous collection and analysis of psychophysiological signals. This method involves measuring physiological parameters as proxies for the activity of a person’s central and autonomic nervous systems to estimate their vigilance level. This approach is based on the hypothesis that the locus coeruleus-norepinephrine system plays a significant role in attention-related activities [13-15]. According to Marois et al [16], this system has been associated with vigilance, attention, orienting, arousal, and the sleep-wake cycle [16-20]. As Marois [16] outlined, several psychophysiological markers of hypovigilance can be gathered using substitute proxy measures of the central nervous system and of the peripheral nervous system. Arslan and Ünal [21] reported that the autonomic nervous system modulates heart rate (HR), blood pressure, digestion, respiration, pupillary reactivity, and regulates other internal functions. Heart rate variability (HRV) is considered a valid measurement for monitoring the autonomic nervous system [22,23], but is not routinely collected in all ICUs.

International guidelines advocate for sedatives and analgesics to ensure patient comfort during painful events [24]. However, when used to induce coma for mechanical ventilation, sedative and analgesic medications such as benzodiazepines and opioids place patients at high risk for delirium [25,26]. According to Riker and Fraser [27], sedative and analgesic therapy is also related to several important side effects, including hypotension, bradycardia and other dysrhythmias, and sepsis. At least one published delirium prediction model includes the use of benzodiazepines and antipsychotics as predictors [3].

Marois et al [16] and Oken et al [28] stated that prediction models using artificial intelligence (AI) have been developed to quantify hypovigilance using psychobehavioral correlates of vigilance in laboratory settings, but real-world examples are lacking. The same scoping review identified 21 psychophysiological models of hypovigilance detection, in which almost all relied on at least one of the following signals, targeting both central and autonomic nervous systems: electrocardiography, photoplethysmography, electroencephalography, electrooculography, and eye tracking [16]. While sensitive, these systems are resource-intensive and hard to use in dynamic environments such as the ICU.

Despite the clinical need for new diagnostic tools, there is still a lack of consensus regarding the most accurate tool to use in clinical practice. There are also significant barriers to the widespread use of more sophisticated diagnostic modalities, such as electroencephalography, in clinical settings due to poor signal quality [16], specialized and costly equipment requirements [29], and patient discomfort associated with extended wear [30]. These factors hinder the adoption of these sensors in routine health care settings. Moreover, other emerging sensors capable of monitoring HRV are not universally incorporated into ICU monitors and are not part of routine data collection.

Patients admitted to the ICU are assessed hourly by critical care nurses to determine their level of vigilance. This assessment is necessary because the condition of patients in the ICU often fluctuates. Current delirium prediction models rely on static baseline data taken at admission, which fail to capture the fluctuating nature of vigilance over time [3]. Moreover, vigilance detection models developed in lab environments lack real-world clinical validation [16]. Clinical real-world settings, such as ICUs, can provide a reliable data collection environment where patients often experience frequent episodes of hypovigilance. Further research is needed to identify effective detection methods for patients in the ICU, including but not limited to the use of automated hypovigilance assessment tools that could be reliably used on a large scale by nonexperts and that could be used in all patients regardless of the language they speak [31]. AI technologies offer a novel modality to support the detection of hypovigilance. The development of a reliable tool capable of monitoring vigilance represents an initial step in the creation of a tool that can accurately diagnose delirium. The objective of this project was to derive an AI-driven prediction model able to continuously detect recurrent episodes of hypovigilance using routinely collected physiological markers in the ICU.

## Methods

### Design and Setting

We conducted a derivation study using data collected from a prospective observational cohort study carried out in the ICU at the Hôtel-Dieu de Lévis Hospital. Its research protocol was not published or registered in a clinical trials registry. We report our findings using the Transparent Reporting of a multivariable prediction model for Individual Prognosis Or Diagnosis for machine learning models (TRIPOD+AI) guidelines [32,33] (checklist provided in Checklist 1). We also used the Prediction model Risk Of Bias Assessment Tool (PROBAST) to identify potential biases [34] (Multimedia Appendix 1). The code and datasets generated to support preprocessing and training the models are available for download from a Zenodo repository [35].

### Participants

Eligible patients were admitted to the Hôtel-Dieu de Lévis Hospital ICU between October 2021 and June 2022. Inclusion criteria were (1) age ≥18 years and (2) an anticipated ICU stay of ≥48 hours from admission. We did not include patients anticipated to stay <48 hours because these patients are often elective patients in the postoperative period undergoing surgeries that require short observation periods in the ICU (eg, simple thoracic surgeries) and carry a much lower risk of developing episodes of hypovigilance during their ICU stay.

Exclusion criteria were (1) inability to obtain informed consent (from patients themselves or their substitute decision-makers), (2) inability to communicate in English or French, (3) neurodegenerative diseases (eg, Alzheimer disease), and (4) unavailability of the data collection device. We excluded patients unable to communicate in English or French or with cognitive disorders, as they would potentially not be capable of answering our study questionnaires, potentially biasing our outcome measure based on the capacity of individuals to interact with bedside nurses. Patients who presented in the ICU when the data collection device was unavailable (eg, due to maintenance or system failure) were not included because no data collection was possible during these periods. We also stopped collecting data for participants who stayed >5 days in the ICU because we wanted to maximize the number of patients included in our study. If we had included data from patients who stayed more than 5 days, all of our team’s human resources would have been spent collecting data on a smaller and less diverse number of patients.

### Data Collection

Despite the inability to blind bedside nurses to the predicted outcome (hypovigilance), they were unaware of the ongoing project. In addition, all vital signs used as predictors were automatically collected by the General Electric (GE) CARESCAPE Gateway, eliminating any potential for information bias.

#### Event Identification

**Figure 1.**
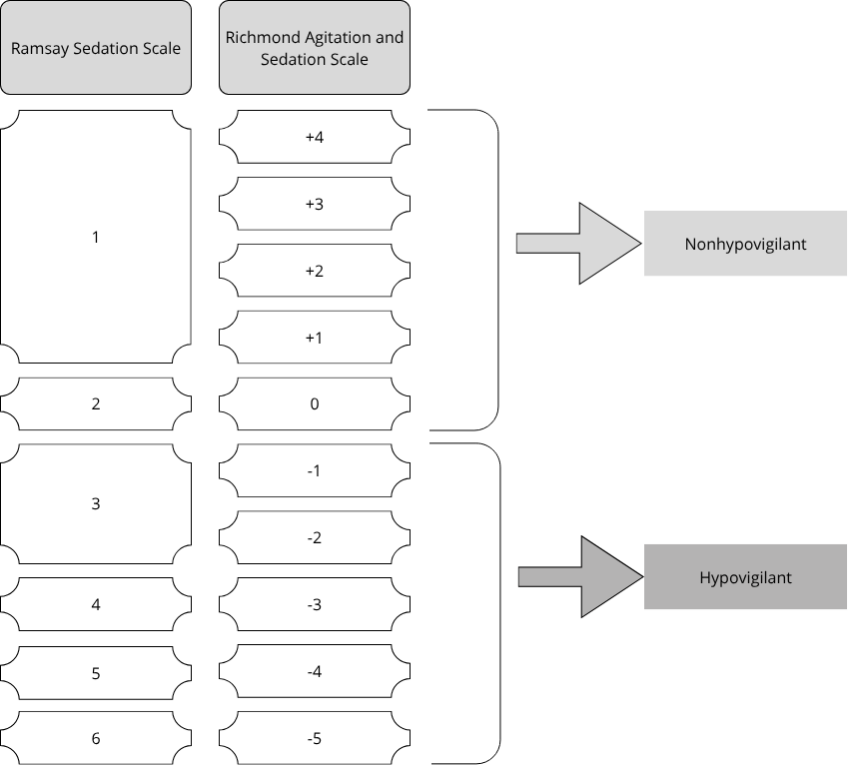
Labeling process to identify episodes of hypovigilance using the corresponding Ramsay Sedation Scale and Richmond Agitation and Sedation Scale.

Intensive care nurses assessed patients’ levels of vigilance using 2 scales. Bedside ICU nurses completed hourly assessments of the patient’s vigilance using the Richmond Agitation and Sedation Scale (RASS) [36] or the Ramsay Sedation Scale (RSS) [37,38]. RASS is a 10-point scale that assesses sedation and agitation based on criteria that evaluate the patient’s response to verbal stimulation. The RSS categorizes sedation levels across 6 states and is widely used in clinical settings [21]. While the RASS is standard for patients who are intubated, the RSS is used when participants are not intubated. There is a strong correlation between the 2 scales, which demonstrates good interrater reliability [39]. Following the study by Mistraletti et al [24], we identified hypovigilant episodes when RASS scores were <0, indicating a drowsy to unarousable state, and RSS scores >2, signifying a drowsy to unarousable condition. These specific criteria served as the basis for labeling vigilance states as episodes of hypovigilance versus nonhypovigilance (Figure 1).

We did not capture the sociodemographic characteristics of the nurses who conducted the RASS and RSS assessments. Vital signs were automatically captured by the GE CARESCAPE Gateway.

#### Clinical Information

At enrollment, we collected participant data on age, sex, height, and comorbidities. We also collected information on the type of admission (medical vs. surgical), history of depression, need for ventilatory support, and need for intubation. We documented whether any intravenously administered sedative or analgesic agents (eg, midazolam, propofol, hydromorphone, or fentanyl) were being administered at the time of vigilance assessment by bedside nurses. Use of intravenous sedation and analgesia was extracted from nursing notes as a binary variable (presence or absence of one of these medications). Multiple medications could be administered simultaneously.

When patients were admitted to the ICU, we also collected data on (1) the Glasgow Coma Scale (GCS) to measure patients’ level of consciousness, ranging from 3 to 15, with lower scores indicating more severe deficits [40,41]; (2) participants’ baseline functional capabilities using the Pfeffer Functional Activities Questionnaire (FAQ) [42]; and (3) the Clinical Frailty Scale (CFS) to evaluate the baseline frailty status of participants with scores ranging from 1 (very fit) to 9 (terminally ill) [43]. These questionnaires are described in Multimedia Appendix 2. These tools were used to describe the population included in our cohort, but were not integrated into our AI algorithm.

#### Physiological Time Series Collection

We used a GE CARESCAPE Gateway (GE HealthCare) to streamline and automate continuous data collection. Gateway data was extracted and securely stored in a comma-separated values format on local servers. Vital signs and physiological markers were continuously monitored and recorded at one-minute intervals, allowing for the exploration of indicators associated with hypovigilant episodes. Bedside vital signs and data automatically recorded via the gateway included HR, respiratory rate (RR), premature ventricular complex count, oxygen saturation, body temperature (when an internal body temperature probe was used), invasive arterial blood pressure, and noninvasive blood pressure (Table 1). Intubation was automatically derived from the presence of inhaled CO_2_ while intubated (CO_2_-IN) or exhaled CO_2_ while intubated (CO_2_-EX; Table 1). Occasionally, more than one timestamp’s worth of data was gathered by the gateway. To ensure data consistency, we systematically removed all duplicate lines.

**Table 1.** Bedside vital signs and data recorded with the General Electric CARESCAPE Gateway.

Feature	Description	Unit
HR	Heart rate	Beats per minute (bpm)
RR	Respiration rate	Breaths per minute (bpm)
SpO_2_-%	Oxygen saturation	Percentage (%)
SpO_2_-R	Pulse oximeter pulse rate	Beats per minute (bpm)
NBP-D	Noninvasive diastolic blood pressure	millimeters of mercury (mm Hg)
NBP-M	Noninvasive mean blood pressure	mm Hg
NBP-S	Noninvasive systolic blood pressure	mm Hg
PVC	Premature ventricular complex count	Events per minute
AR-D	Arterial line diastolic pressure	mm Hg
AR-S	Arterial line systolic pressure	mm Hg
AR-M	Arterial line mean pressure	mm Hg
AR-R	Arterial line pulse rate	Beats per minute (bpm)
CO_2_-EX	Exhaled CO_2_ while intubated	mm Hg
CO_2_-IN	Inhaled CO_2_ while intubated	mm Hg
Temperature	Rectal temperature	Degrees Celsius (°C)

### Time Series Selection

**Figure 2.**
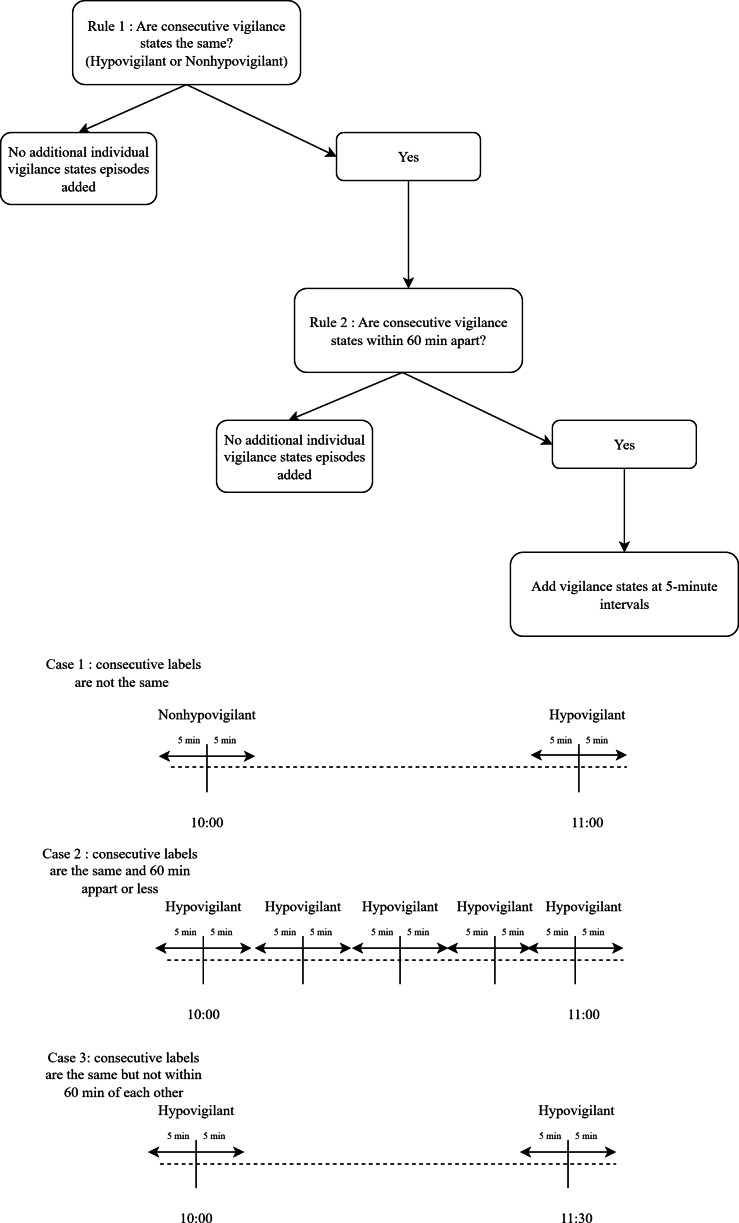
Label identification process.

Time series of sequential changes in vigilance states were generated by selecting physiological measurement data within a 5-minute window before and after each hypovigilant or nonhypovigilant episode, resulting in an 11-point time series spanning 11 minutes (Figure 2). RASS and RSS assessments were made hourly; if two measurement points were the same, the condition was considered constant throughout the hour. When two consecutive vigilance levels were different, no assumptions were made for the time points between these two vigilance assessments. The decision to use an 11-minute window in each time series aimed to maximize clinical relevance, better characterize state changes, and optimize the classification capacity of our AI models.

Figure 2 illustrates the 2 simple rules we followed to label episodes of hypovigilance before and after the hourly vigilance assessments determined by bedside nurses. Labels (hypovigilant vs nonhypovigilant) were automatically assigned for each separate vigilance state as determined by bedside nurses. Additional imputed labels were assigned to time points before and after each hourly vigilance assessment performed by the nurses based on two simple rules. The first rule determined if two labels were within 60 minutes of each other. If two consecutive vigilance assessments were made ≤60 minutes apart, we proceeded to the second rule. If the assessments’ labels were >60 minutes apart, we did not add any new labels for new hypovigilance episodes. The second rule determined if the consecutive vigilance states (and associated labels) were identical. If they were identical, we added labels at 5-minute intervals for each episode of hypovigilance or nonhypovigilance between the original labels, based on the value of the vigilance state at both boundaries. If they were not identical, we did not add additional episodes of hypovigilance or nonhypovigilance between the consecutive discordant labels, because determining the moment when the state changed from hypovigilant to nonhypovigilant (or vice versa) was not documented by bedside nurses and was impossible to determine retrospectively. For example, in case 1, no additional labels were added because the consecutive labels differed. In case 2, labels were added at 5-minute intervals when the consecutive labels were identical and less than 60 minutes apart. However, in case 3, no additional labels were added because the consecutive states remained the same but were separated by more than 60 minutes. This approach preserved the temporal structure integrity of our model.

### Time Series Preprocessing

#### Missing Values and Data Cleaning

We used a backward- and forward-filling strategy to address missing values in the vital signs time series [44]. Backward filling involves filling in missing values in a dataset by using preceding data values to complete the gaps. In other words, missing values were filled based on available data preceding them in the time series [44]. In some cases, backward filing was impossible due to the lack of available previous data. In such cases, forward filling was performed. Forward filling involves using future values to fill in missing data [44].

Real-world data is also inevitably contaminated with noise, artifacts, and unreliability due to patient movement, sensor unavailability, or electrical interference. We deliberately chose to analyze all of the data, knowing that it may present noise and artifacts, using robust machine learning techniques instead of relying solely on cleaned datasets for traditional statistical analysis. We aimed to develop a model that is not only representative of real-world conditions but also capable of generalizing to diverse clinical scenarios. By adopting a machine learning approach, we could effectively learn from the inherent variability in the data and sensor availability, including occasional artifacts, rather than eliminating them entirely. Excessive data cleaning and artifact removal may result in a model that performs well in a controlled setting but fails to translate effectively in real-world applications. Our methodology emphasizes the importance of building resilience in our models to account for the inevitable noise present in ICU data.

#### Features Extraction

Since the objective of this project was to derive an AI algorithm capable of continuously detecting recurrent episodes of hypovigilance using routinely collected physiological markers in the ICU, we focused our development on physiological features that could be automatically captured by the GE CARESCAPE Gateway. As features for our model, we extracted the first-, second-, and third-order derivatives for each participant’s temporal data stream to observe global variations or trends across all patient observations. The first derivative represents the rate of change over time. For example, it allows for the identification of rapidly increasing or decreasing blood pressure. The second derivative refers to the acceleration of the rate of change, for example, how the slope of a vital signs curve evolves over time [45]. A positive second derivative might suggest an acceleration in blood pressure increase, while a negative second derivative could indicate an acceleration in blood pressure decrease. The third derivative captures variation in acceleration, that is, the rate at which the acceleration changes [46]. This third derivative can be useful for detecting unusual changes. By using derivatives, we aimed to capture subtle physiological changes over time that might otherwise go unnoticed.

We also observed that some features (arterial line diastolic pressure, arterial line mean pressure, arterial line pulse rate, arterial line systolic pressure, temperature, CO_2_-EX, and CO_2_-IN) were missing for >40% of participants. This can be explained because these sensors were only used in certain critically ill patients when indicated. The presence of these features in certain patients reflects that a patient is critically ill and needs more invasive life support (eg, intubation, mechanical ventilation, and sedation) or invasive monitoring (eg, arterial blood pressure catheter or internal temperature probe). CO_2_-EX and CO_2_-IN are features only available when a patient is intubated. The presence of a temperature measurement captured by the GE CARESCAPE Gateway is only available when an internal temperature probe is used. Variables measured by an arterial line (arterial line diastolic pressure, arterial line mean pressure, arterial line pulse rate, and arterial line systolic pressure) are only available when an arterial line is installed. To mitigate potential bias in our classifier due to missing variables in less ill patients, we replaced these features with Boolean (presence or absence) variables indicating whether an arterial line was present, the patient was intubated, or an internal body temperature probe was used. This approach enhances the generalizability of our findings by accurately representing typical ICU practices.

, arterial line mean pressure, arterial line pulse rate, and arterial line systolic pressure) are only available when an arterial line is installed. To mitigate potential bias in our classifier due to missing variables in less ill patients, we replaced these features with Boolean (presence or absence) variables indicating whether an arterial line was present, the patient was intubated, or an internal body temperature probe was used. This approach enhances the generalizability of our findings by accurately representing typical ICU practices.

#### Features Selection

**Figure 3.**
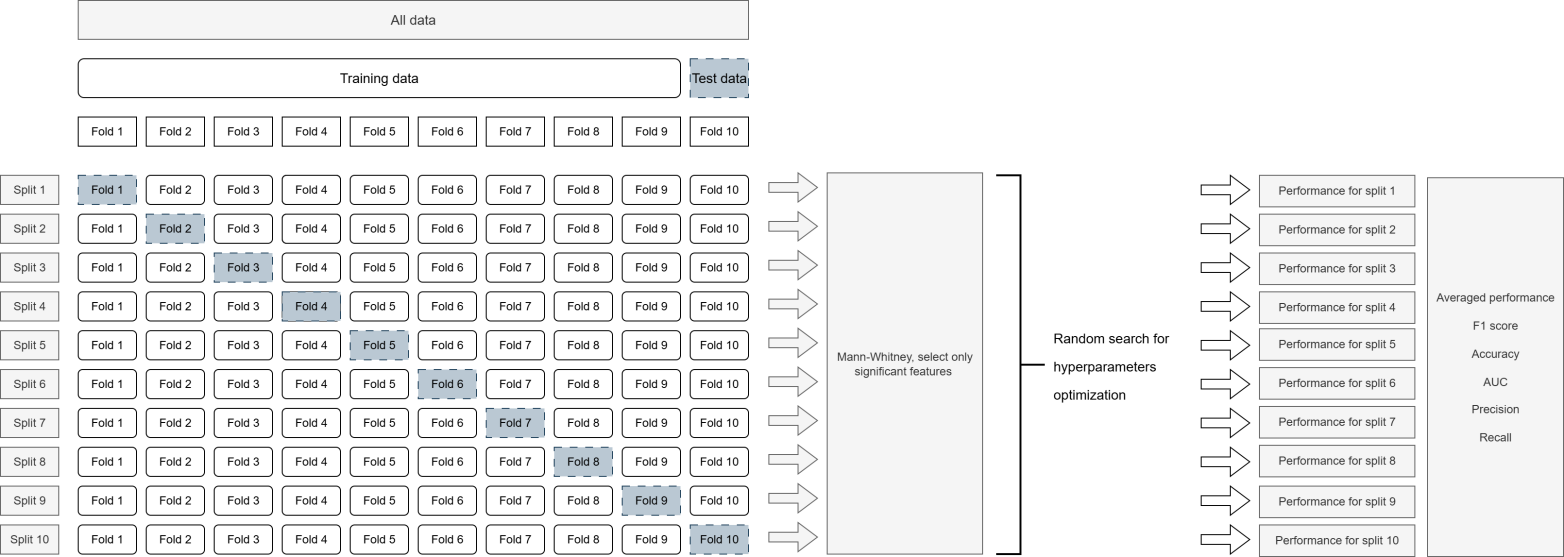
Data splitting and hyperparameter optimization to evaluate the performance of the models. AUC: area under the curve.

Our objective was to identify significant differences between the two vigilance states (hypovigilant vs nonhypovigilant) with a non-normal distribution of the data. We elected to reduce the feature space by only selecting features that were significantly different between the two states, on average. To this end, we performed Mann-Whitney *U* tests [47] on the distribution of the features. The dataset was divided into training and test sets (refer to Figure 3), and the Mann-Whitney *U* test was performed separately on each set. Only the variables that were statistically significant within a particular set were included in the respective model trained on that set. As a result, multiple models were generated, each using a subset of the variables found to be significant in their respective sets. This approach ensured that the models were tuned to capture the most relevant features for predicting hypovigilance states, considering the variability observed across sets during the cross-validation process. To provide statistics on the selected features, we counted the number of times a variable was found to be significant across sets.

### Machine Learning Models

We used 3 distinct AI models for detecting hypovigilance events: Random forest (RF) from the Python Scikit-learn library [48], Extreme Gradient Boosting (XGBoost) from the XGBoost library [49], and the Light Gradient Boosting Machine (LightGBM) classifier from the LightGBM library [50]. We chose these classifiers because RF and XGBoost were used in prior studies to identify delirium and hypovigilance [28]. LightGBM was also used because of its previous application in other ICU databases, such as the Medical Information Mart for Intensive Care III database [51].

RF is a real-time classification algorithm that excels in capturing nonlinear relationships, making it applicable to domains such as clinical outcome prediction [52]. It is composed of a set of data structures characterized by decisions (branches), called trees, with each tree depending on random variables. It creates a forest from a group of decision trees trained using the bagging method. The key notion behind the bagging method is the combination of multiple learning models to improve overall sensitivity [52]. XGBoost uses gradient-based decision trees. It is tailored for classification and regression modeling of tabular datasets [49]. As described by Qian et al [51], the LightGBM classifier uses iterative training to obtain the most advantageous identification model. Qian et al [51] explained that LightGBM uses a gradient-boosting framework using a tree-based learning algorithm to reduce computation time.

### Data Splitting and Hyperparameters Search

To preserve patient data and account for the limited number of patients in our dataset, we used a 10-fold, group-based cross-validation strategy. The data were partitioned into groups of random size at the patient level. This approach ensured that all within-patient-related information was retained during model evaluation, making models more robust to new participants.

To enhance the performance of our 3 AI classifiers, we used the random search technique for hyperparameter tuning for each split. This technique is widely recognized for its computational efficiency compared to traditional grid search methods, as it requires less computational time [53].

In Figure 3, the model was trained and evaluated using group cross-validation. The data were split at the patient level into training and testing subsets for each fold of the cross-validation, with varying group sizes ranging from one to several patients. Across all folds, the Mann-Whitney U test was performed to select only the significant features in each model. Performance metrics (accuracy, precision, and recall) were computed on the testing subset during each fold.

### Performance Evaluation

The performance metrics included average accuracy, which measured the proportion of correct predictions made by the model across all iterations of the cross-validation process; precision, which assessed the proportion of true positive predictions among all positive predictions, providing insight into the ability of the model to make precise classifications; and average recall (sensitivity), which evaluated the capability of the model to correctly identify positive instances from the entire dataset. In addition, we computed the average area under the curve (AUC), which serves as a measure of the model’s ability to distinguish between positive and negative classes, and the average *F*_1_-score, which provides a balanced assessment by considering both precision and recall. These metrics collectively indicate the overall performance of our model in classification tasks. We also generated calibration curves for the 3 different classifiers using one representative model from the cross-validation process.

To interpret the predictions of our best-performing models, we conducted a Shapley Additive Explanations (SHAP) analysis [54]. For this analysis, we selected one of the top-performing models identified during 10-fold group-based cross-validation and retrained it on the entire dataset of 30 participants. The resulting SHAP values provided insight into each feature's contribution to the model's output, although the exact feature importance may vary between individual model.

### Sensitivity Analyses

We also aimed to evaluate the impact of including the medication variable (use of sedatives or analgesics) on the performance of our models. We therefore performed 2 main sets of sensitivity analyses: one excluding and the other including the medication variable as a variable of interest.

### Patient and Public Involvement

Patients and the public were not involved in the design, conduct, reporting, or dissemination plans of this research.

### Ethical Considerations

The study was approved by the research ethics committee of the Centre intégré de santé et de services sociaux de Chaudière-Appalaches (2021‐771).

## Results

### Patient Characteristics

**Figure 4.**
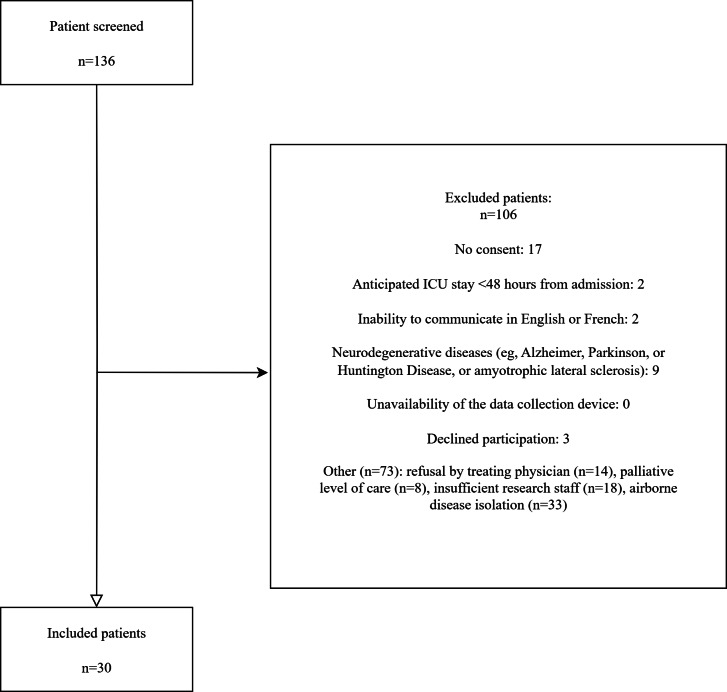
Flowchart of the data collection process. ICU: intensive care unit.

Among 136 patients considered for inclusion in our cohort, 30 were eligible (Figure 4). These 30 patients experienced a total of 1493 hypovigilant episodes and 764 nonhypovigilant episodes. Two participants did not have any hypovigilant episodes. As shown in Table 2, participants in our cohort were aged 69 years (mean), male (63%), admitted to the ICU for surgical (30%) or medical (70%) reasons, and mostly intubated, receiving intravenous sedation-analgesia medication (70%).

**Table 2.** Demographic and clinical characteristics of participants (N=30).

Characteristic	Value
Age (years), mean (SD); range^a^	68.9 (11.0); 35‐86
Height (cm), mean (SD); range^a^	168.0 (8.9); 152‐183
Sex, n (%)	
Women	11 (36.7)
Men	19 (63.3)
Length of ICU^b^ stay (days), mean (SD); range^a^	8.60 (5.29); 1.33‐22.24
Comorbidities, n (%)	
Cardiovascular diseases	22 (73.3)
Respiratory disease	14 (46.7)
Renal disease	8 (26.7)
Diabetes	8 (26.7)
History of stroke	3 (10.0)
No comorbidities	1 (3.3)
Other	21 (70.0)
Depression in the past, n (%)	
Yes	1 (3.3)
No	29 (96.7)
Type of admission, n (%)	
Medical	21 (70.0)
Surgical	9 (30.0)
Respiratory assistance, n (%)	
Yes	25 (83.3)
No	5 (16.7)
Intubated, n (%)	
Yes	21 (70.0)
No	9 (30.0)
Admission assessment	
Glasgow Coma Scale, mean (SD); range^a^	14 (1); 8‐15
Functional Activity Questionnaire (FAQ), mean (SD); range^a^	3 (5); 0‐19
Clinical Frailty Scale, median (IQR^c^)	3 (2-4)

a Range: minimum - maximum values.

b ICU: intensive care unit.

c IQR: interquatile range.

**Figure 5.**
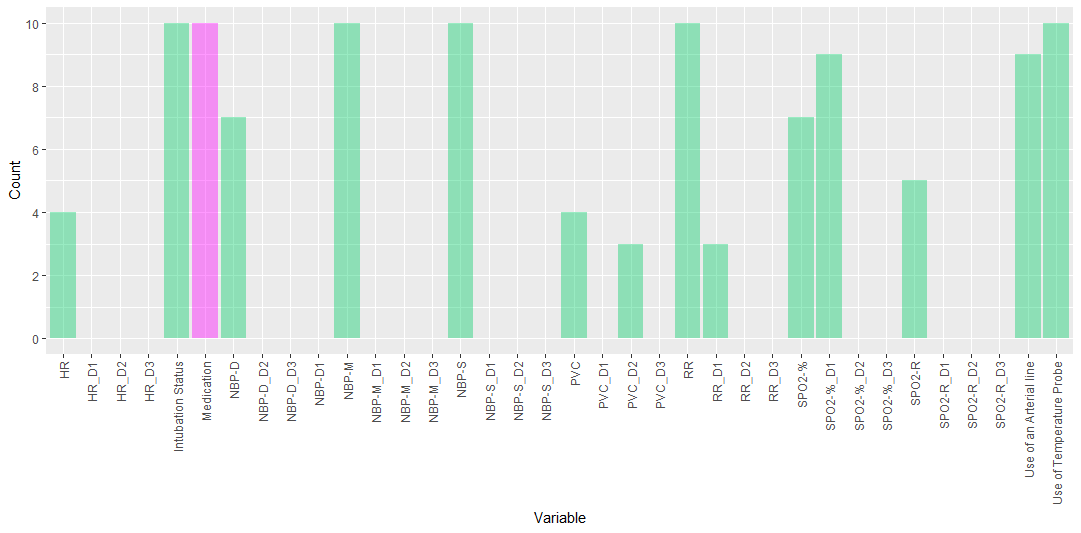
Significant features in the models. Green bars represent features identified as significant across the 10-fold cross-validation procedure using the base model without the inclusion of the medication variable. The purple bar indicates the addition of the medication variable, which was consistently selected as a significant feature across all folds.

Figure 5 illustrates the frequency of significant features identified through the Mann-Whitney *U* test conducted during the LightGBM cross-validation. This is consistent across all classifiers, as they all used the same groups. Green bars represent features identified as significant across the 10-fold cross-validation procedure using the base model without the inclusion of the medication variable. The purple bar indicates the addition of the medication variable, which was consistently selected as a significant feature across all folds. The first, second, and third derivatives are labeled as “_D1,” “_D2,” and “_D3,” respectively.

Variables that were consistently significant across multiple folds were intubation, noninvasive mean blood pressure, noninvasive systolic blood pressure, RR, and the presence of an internal body temperature probe. Other key features were also frequently found significant included the presence of an arterial line, noninvasive diastolic blood pressure, premature ventricular complex count (PVC) count, the second derivative of PVC count (PVC_D2), the first derivative of RR (RR_D1), oxygen saturation (SpO_2_-%), and HR determined by the pulse oximeter pulse rate. These features exhibited varying degrees of importance across cross-validation folds, suggesting their potential relevance in detecting hypovigilance episodes. Notably, the use of intravenous sedation and analgesia medication variable was significant in 10 instances, highlighting its importance in our models. In addition, only the following derivatives were significant in our feature analysis: PVC_D2, RR_D1, and SpO_2__D1.

### Classification Results

The classification results of the three AI classifiers—XGBoost, RF, and LightGBM—along with an additional set of the 3 models incorporating the sedative or analgesic medication variable, are presented in Table 3.

For the models excluding the sedative or analgesic medication variable, the LightGBM model demonstrated the highest average accuracy, average precision, average recall, average AUC, and average *F*_1_-score. Furthermore, it exhibited an average recall of 74% (SD 18%) and an average precision of 76% (SD 11%). XGBoost followed as the second-best classifier with an average recall of 73% (SD 18%) and an average precision of 75% (SD 10%). When the sedative or analgesic medication variable was incorporated, LightGBM remained the top-performing classifier, closely followed by XGBoost and RF. Their performances are relatively similar, except in terms of average recall, where both XGBoost and LightGBM achieved 70% and 71%, respectively, and RF achieved 64%.

**Table 3.** Classification performance metrics for our 3 artificial intelligence models.

Model	Average accuracy, mean (SD)	Average precision, mean (SD)	Average recall, mean (SD)	Average AUC^a^, mean (SD)	Average *F*_1_-score, mean (SD)
Models without incorporating the sedative or analgesic medication variable					
XGBoost^b^	0.66 (0.11)	0.75 (0.10)	0.73 (0.18)	0.58 (0.09)	0.68 (0.10)
Random forest	0.67 (0.15)	0.76 (0.11)	0.68 (0.22)	0.60 (0.12)	0.69 (0.12)
LightGBM^c^	0.68 (0.12)	0.76 (0.11)	0.74 (0.18)	0.60 (0.12)	0.69 (0.11)
Models with the incorporation of the sedative or analgesic medication variable					
XGBoost	0.70 (0.15)	0.76 (0.13)	0.70 (0.19)	0.62 (0.14)	0.72 (0.14)
Random Forest	0.71 (0.15)	0.77 (0.13)	0.64 (0.25)	0.63 (0.13)	0.72 (0.15)
LightGBM	0.70 (0.15)	0.76 (0.13)	0.71 (0.21)	0.62 (0.12)	0.72 (0.14)

a AUC: area under the curve.

b XGBoost: Extreme Gradient Boosting.

c LightGBM: Light Gradient Boosting Machine.

### Feature Importance

To better understand which physiological parameters most influenced the model’s prediction of hypovigilance, we computed SHAP values for the features used in our LightGBM classifier. The analysis revealed that intubation status, noninvasive systolic blood pressure, and RR were the most influential predictors. A detailed SHAP summary plot is provided in the Multimedia Appendix 3. For this specific model, the first derivative of oxygen saturation (SpO_2_-%_D1) also had an impact. This indicates that the rate of change in oxygen saturation appears to be more informative in assessing vigilance than the absolute oxygen saturation level itself. No other derivatives influenced the model presented.

**Figure 6.**
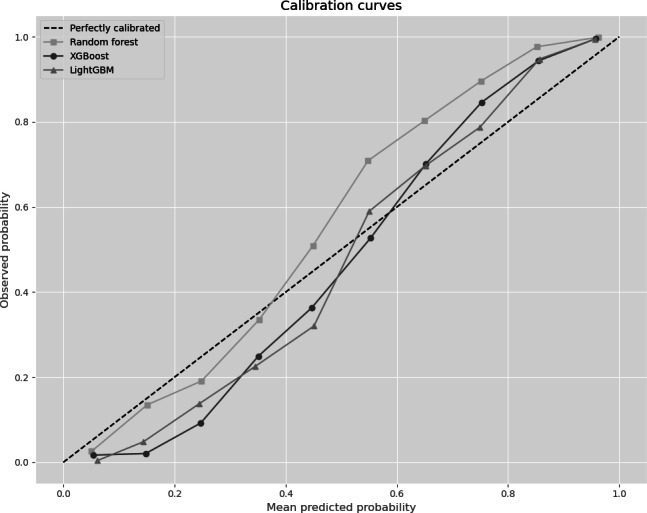
Calibration curves comparison. LightGBM: Light Gradient Boosting Machine; XGBoost: Extreme Gradient Boosting.

Figure 6 presents calibration curves comparing 3 classifier models. The black dotted diagonal line represents ideal calibration, where predicted probabilities perfectly align with observed event frequencies. Deviations above the diagonal indicate overestimation of probabilities (overconfidence), and deviations below indicate underestimation (underconfidence). In this specific test fold, the RF model (line with squares) displayed the most substantial deviations, indicating the least accurate calibration among the classifiers. The XGBoost model (line with circles) exhibited improved calibration, albeit with some residual discrepancies. The LightGBM model (line with triangles) demonstrated the closest approximation to perfect calibration. It is important to note that the cross-validation procedure resulted in 30 distinct models, and this figure illustrates only 3 representative examples. Although this study’s primary objective was to evaluate predictive performance, future research should prioritize calibration techniques to optimize probability estimates in the final selected model.

## Discussion

### Principal Findings

This study aimed to develop an AI model to identify hypovigilance episodes in patients using data from a single ICU. Our results demonstrate that the differentiation of episodes of hypovigilance from nonhypovigilance episodes is possible with 3 different classifiers using routinely acquired clinical ICU data.

While most researchers agree that an AUC below 0.6 indicates poor performance, there is less consensus on how to classify higher values [55]. AUC values between 0.7 and 0.8, for example, have been inconsistently described as poor, moderate, fair, or even good. We acknowledge that the performance statistics of our AI models, ranging in the poor to moderate range (AUCs of 0.58-0.63), preclude any clinical application at this time.

The LightGBM classifier showed slightly better results than XGBoost and RF across multiple evaluation criteria. However, all the classifiers showed significant variability in correctly identifying true positives across different folds of the cross-validation process. The high SD of our results may be due to differences in participant characteristics or class imbalances during training. A precision score of 76%, with a SD of 11%, indicates the ability to correctly identify true positives among all positive results across different folds. LightGBM classifiers generally outperformed both XGBoost and RF in terms of average accuracy, precision, recall, AUC, and *F*_1_-score. These results demonstrate the need for further refinement and prospective, external validation with larger datasets. The XGBoost algorithm achieved a recall rate of 73% and an average precision rate of 75%. A screening tool needs to be sensitive and have a high recall [56]. Our average recall rate of 74% indicates we still need to decrease the number of false negatives generated by our model, because missing an episode of hypovigilance in the ICU could have serious consequences. Our sensitivity analysis revealed a clear improvement in model performance with the inclusion of medication data about the use of intravenous sedatives and analgesics, which correlated with the occurrence of hypovigilant episodes. This suggests the importance of incorporating other time-dependent concurrent contextual data into a predictive model developed to continuously monitor for the risk of hypovigilance.

Although our models do not demonstrate performance characteristics to support current clinical application, they collectively demonstrate promise for potential future refinement and research. Despite these limitations, our models remain superior to random chance and offer the opportunity to inform future studies on patients whose level of vigilance is at risk of fluctuating in the ICU. Future studies on this subject need to ascertain whether performance can be enhanced over time, including the calibration of a future model to optimize probability estimates. Any AI tool that enhances patient care, particularly for those who are most vulnerable in the ICU, is worthy of further investigation and validation if conducted ethically and with respect for equity, patient privacy, high-quality standards, and transparent data reporting.

### Comparison With Prior Work

The majority of research on hypovigilance has previously been conducted in laboratory settings, which offer a highly regulated setting but might not accurately reflect real-world ICU situations [16]. Also, the field of vigilance is often poorly defined, which makes it hard to compare our results to the existing literature [28]. The existing vigilance research is mostly focused on driving and flight simulations, during which operators do experience hypovigilance, but it may not present the same as in patients in the ICU [16].

Although our study did not focus on the detection of delirium, we did study how to detect hypovigilance, which is an important component of hypoactive delirium. Comparable delirium prediction models, which also use noninvasive features, show similar or slightly better performances. For example, a model developed in South Korea by Oh et al [57], using automatically collected variables including HRV, achieved a slightly better-balanced accuracy of 70%, with a maximum accuracy of 71.5%. Despite its lower performance, our model still shows promise considering that we derived our algorithm on a smaller dataset without HRV data. These results are also in line with other delirium studies using electroencephalography and electrocardiography [58].

To improve the accuracy of future delirium diagnostic and prediction models, we considered the dynamic nature of a patient’s condition and incorporated real-time data into our models that centered only on the detection of hypovigilance and not delirium [3]. A future enhanced and more accurate automated model could potentially offer real-time patient monitoring throughout their ICU stay. Such a model could use data that are generally accessible across all ICUs. As mentioned by Marois et al [16], the variability of the baseline “gold standard” in hypovigilance prediction models is a significant challenge. Different studies use diverse gold standards, some lacking prior validation. To address this, our study used 2 validated sedation scales (RSS and RASS) in a clinical setting, incorporating a validated gold standard to enhance the labeling process of hypovigilant episodes.

### Strengths

Our study has several strengths. First, by conducting our research in a real-world ICU setting and using simple and routinely collected vital signs and physiological markers used in ICUs around the world, we ensured that our findings are relevant and transferable to similar health care settings. In addition, we used rigorous data collection methods using an automated vital sign data collection system. This ensured the consistency and accuracy of our dataset, minimizing the risk of classification bias. Our data collection and classification methods are entirely noninvasive and exclude procedures such as blood tests or electroencephalography, thereby increasing the integration potential into AI-based decision support systems. Given that no baseline sociodemographic variables such as age, sex, past medical history, or other clinical variables were included in our model, our first model without the intravenous sedative or analgesic medication variable could stand alone without any human-collected data. Another strength of our model is that our AI-derived detection model based on automatically collected vital signs is agnostic to language, which makes it more equitable for patients who speak languages different from their health care providers, and in settings where hypovigilance and delirium are assessed using detection tools that depend on understanding the language being used.

Our study analyzed numerous episodes of hypovigilance and nonhypovigilance in patients in the ICU. These episodes often lasted for extended periods, making them suitable for detailed analysis. The regular assessments of vigilance by experienced nurses using the RASS or RSS provided a rich dataset for training machine learning models. The expertise and familiarity of the bedside nurses with these standard assessment tools contributed to the reliability and credibility of our outcome measures.

Our study also took advantage of the routine use of intravenous sedatives and analgesics in the ICU, such as propofol, hydromorphone, fentanyl, and midazolam, that induce prolonged states of deep sedation. This provided valuable opportunities to detect episodes of hypovigilance, allowing us to refine our model and improve its effectiveness in identifying clinically relevant conditions. Identification of hypovigilance has important implications for health care settings in screening persons at risk of delirium. Delirium screening is a time-consuming task that requires completing multicomponent screening tools such as the Confusion Assessment Method for the ICU. Motivated by the growing sophistication of AI models in the medical domain, our project investigated the possibility of using machine learning to enhance the screening capacity of hypovigilance in the context of nursing shortages. This potentially represents a viable path toward improving patient outcomes and decreasing the workload of health care professionals [59]. The creation of AI algorithms capable of detecting early onset of hypovigilant episodes may allow clinicians to apply timely delirium treatments or mitigation measures, thereby enhancing the outcomes of patients in the ICU.

### Limitations

We used the PROBAST checklist to assess the risk of bias in our models (Multimedia Appendix 1) [34]. Based on this assessment, we identified several limitations to our study. First, our study recruited a small cohort of 30 participants and only used data collected for the first 5 days of their ICU stay. Data collection was limited to 5 days because we wanted to maximize the number of patients included in the study. If we had included data from patients during their entire ICU stay, we would have risked biasing our results with some patients who can stay up to several months in the ICU. In such cases, human resources would have been spent on collecting data for fewer patients, thus threatening the external validity of our study. Moreover, obtaining consent for studies in critical care settings can be difficult because substitute decision-makers are not always available. Even though we had a small number of individual participants, each participant underwent hourly, 24-hour vigilance assessments. In addition, the GE CARESCAPE Gateway recorded vital signs at 1-minute intervals, yielding a substantial amount of data for each participant. To address the limitation associated with the small size of our cohort, we used a 10-fold group cross-validation approach. Future studies that include a larger sample in other ICUs will help identify new patterns in physiological marker fluctuations that will help identify hypovigilant states. The cross-validation method used to evaluate the performance of our model across multiple patient groups helped us make full use of our small dataset. The cross-validation strategy also helped identify stable and reliable model performance metrics, minimizing overfitting risk and providing more accurate estimates of the true performance of our model on the entire patient dataset. The wide SDs of our performance metrics are attributed to our small dataset. Nonetheless, our models showed moderate discriminative power, surpassing chance, which suggests a hopeful path for future refinement and improvement. Future studies will need to include a greater number of participants to allow stratification based on comorbidities and detect within-class trends. This effort would require a deep learning approach to deal with the high number of potential interaction terms between different comorbidities and the risk of hypovigilance. Moreover, a larger sample size would allow a future AI model to produce a more powerful and better-calibrated model capable of predicting discrete ordinal outcomes (eg, discriminating between a RASS of 0 vs –1 [drowsy] vs –2 [light sedation] or –3 [moderate sedation]).

A second limitation comes from the fact that our models were built using physiological data captured at low frequency, using 1-minute intervals. Low-frequency data collection limited our ability to capture the subtle changes in high-frequency variations that could be manifested in the transition from nonhypovigilant to hypovigilant states, such as changes in HRV. HRV would have been a valuable characteristic, as shown in other contexts [16], but we could not collect this data with our current GE CARESCAPE Gateway setup. To address this lack of HRV, we investigated the use of the derivatives of the HR variable in our study to measure the rate of change of HR in our model, as well as the use of other cardiac measures such as PVC count as a surrogate for heart irritability and sympathetic nervous system activation [60]. Despite this effort, we did not find a relationship between the derivatives of the HR and the occurrence of hypovigilance. We did find, however, that the rate of change of oxygenation saturation (first derivative of SpO_2_%) did predict hypovigilance, which may have some biological plausibility because lower saturation leads to lower brain tissue oxygenation [61].

Third, while other more sophisticated hypovigilance detection models incorporate continuous electroencephalography data [28], this was not possible in our study. Few ICUs have access to continuous electroencephalography monitoring simultaneously for all their patients, underscoring the importance of developing a model that does not rely on these rarely available sensors to ensure its generalizability to many settings. Hence, our approach enables the detection of hypovigilance in a broader context, where electroencephalography may not be readily available.

Fourth, our exclusion of patients with cognitive deficits limits the external validity of our findings. Any future refinements of our AI model will need to include these high-risk populations who are increasingly becoming frequent patients in the ICU [62]. Despite this limitation, our study adds evidence about the feasibility of conducting a privacy-compliant and ethically responsible AI study with vulnerable patients in the ICU that holds promise to improve the quality of patient care in the ICU.

Finally, we did not include patient comorbidities or elements from the medical history in our model development. Including these additional features could have potentially resulted in a superior model, but the decision was made to exclude them because our objective was to develop an automated tool that would minimize the burden on busy clinicians and rely only on features automatically captured by the GE CARESCAPE Gateway. Despite this, we did explore whether using data about sedative and analgesic medication administered would improve the performance of our model, even if medication administration is manually documented by bedside nurses in patients’ charts. Future studies will have to explore how to automatically capture and integrate data about the administration of psychoactive medications, their administration route, their exact time of administration, and their interaction with any preexisting comorbidities. Other potential candidate predictor variables could also be included to enhance the performance of future AI models, such as the time of the day [63] or ambient noise level in the room [64].

### Conclusion

We developed an automatic machine learning algorithm to detect hypovigilance in patients in the ICU using routine and easily captured physiological parameters. The classifiers presented in this study demonstrated that hypovigilance could be distinguished from nonhypovigilance cases with poor-to-modest results. Our models exhibited potential for future improvement. Our study adds to the increasing evidence about the potential of machine learning algorithms in real-world clinical settings and identifies avenues for future research to enhance the detection of hypovigilance and improve patient outcomes.

## Supplementary material

10.2196/60885 Multimedia Appendix 1 PROBAST (Prediction model Risk Of Bias Assessment Tool) to identify potential biases.

10.2196/60885 Multimedia Appendix 2 Questionnaires used to describe the cohort.

10.2196/60885 Multimedia Appendix 3 Shapley Additive Explanations (SHAP) values of the Light Gradient Boosting Machine model without excluding the sedative or analgesic medication variable.

10.2196/60885 Checklist 1 Transparent Reporting of a multivariable prediction model for Individual Prognosis Or Diagnosis for machine learning models (TRIPOD+AI) checklist.

## Abbreviations

AI: artificial intelligence
AUC: area under the curve
CFS: Clinical Frailty Scale
CO2-EX: exhaled CO2 while intubated
CO2-IN: inhaled CO2 while intubated
*DSM-5*: *American Psychiatric Association’s Diagnostic and Statistical Manual of Mental Disorders, Fifth Edition*
FAQ: Functional Activities Questionnaire
GCS: Glasgow Coma Scale
GE: General Electric
HR: heart rate
HRV: heart rate variability
ICU: intensive care unit
LightGBM: Light Gradient Boosting Machine
PROBAST: Prediction model Risk Of Bias Assessment Tool
PVC: premature ventricular complex count
RASS: Richmond Agitation and Sedation Scale
RF: random forest
RR: respiratory rate
RSS: Ramsay Sedation Scale
SHAP: Shapley Additive Explanations
SPO2-%: oxygen saturation
TRIPOD+AI: Transparent Reporting of a multivariable prediction model for Individual Prognosis Or Diagnosis for machine learning models
XGBoost: Extreme Gradient Boosting
