# Supplementary material for: Predicting Episodes of Hypovigilance in Intensive Care Units Using Routine Physiological Parameters and Artificial Intelligence: Derivation Study
Source: JMIR AI. 2025 Aug 27;4:e60885. doi: 10.2196/60885 (PMC12384691; doi:10.2196/60885)
Supplement: Multimedia Appendix 1 [file ai-v4-e60885-s001.docx]

**PROBAST**

(Prediction model study Risk Of Bias Assessment Tool)

Published in Annals of Internal Medicine (freely available):

1. [PROBAST: A Tool to Assess the Risk of Bias and Applicability of Prediction Model Studies](https://annals.org/aim/fullarticle/2719961/probast-tool-assess-risk-bias-applicability-prediction-model-studies)
2. [PROBAST: A Tool to Assess Risk of Bias and Applicability of Prediction Model Studies: Explanation and Elaboration](https://annals.org/aim/fullarticle/2719962/probast-tool-assess-risk-bias-applicability-prediction-model-studies-explanation)

| **Step** | **Task** | **When to complete** |
| --- | --- | --- |
| **1** | Specify your systematic review question(s) | Once per systematic review |
| **2** | Classify the type of prediction model evaluation | Once for each model of interest in each publication being assessed, for each relevant outcome |
| **3** | Assess risk of bias and applicability | Once for each development and validation of each distinct prediction model in a publication |
| **4** | Overall judgment | Once for each development and validation of each distinct prediction model in a publication |

**What does PROBAST assess?**

PROBAST assesses both the *risk of bias* and *concerns regarding applicability* of a study that evaluates (develops, validates or updates) a multivariable diagnostic or prognostic prediction model. It is designed to assess primary studies included in a systematic review.

*Bias* occurs if systematic flaws or limitations in the design, conduct or analysis of a primary study distort the results. For the purpose of prediction modeling studies, we have defined *risk of bias* to occur when shortcomings in the study design, conduct or analysis lead to systematically distorted estimates of a model’s predictive performance or to an inadequate model to address the research question. Model predictive performance is typically evaluated using calibration, discrimination and sometimes classification measures, and these are likely inaccurately estimated in studies with high risk of bias. *Applicability* refers to the extent to which the prediction model from the primary study matches your systematic review question, for example in terms of the participants, predictors or outcome of interest.

A primary study may include the development and/or validation or update of more than one prediction model. A PROBAST assessment should be completed for each distinct model that is developed, validated or updated (extended) for making individualised predictions. Where a publication assesses multiple prediction models, only complete a PROBAST assessment for those models that meet the inclusion criteria for your systematic review. Please note that subsequent use of the term “model” includes derivatives of models, such as simplified risk scores, nomograms, or recalibrations of models.

PROBAST is not designed for all multivariable diagnostic or prognostic studies. For example, studies using multivariable models to identify predictors associated with an outcome but not attempting to develop a model for making individualised predictions are not covered by PROBAST.

PROBAST includes four steps.

If this is your first time using PROBAST, we strongly recommend reading the detailed explanation and elaboration (E&E, see link above) paper and to check the examples on www.probast.org

# Step 1: Specify your systematic review question

State your systematic review question to facilitate the assessment of the applicability of the evaluated models to your question. *The following table should be completed once per systematic review.*

| **Criteria** | **Specify your systematic review question** |
| --- | --- |
| *Intended use of model:* | N/A |
| ***Participants*** *including selection criteria and setting:* | *N/A* |
| ***Predictors*** *(used in prediction modeling), including types of predictors (e.g. history, clinical examination, biochemical markers, imaging tests), time of measurement, specific measurement issues (e.g., any requirements/ prohibitions for specialized equipment):* | N/A |
| *Outcome to be predicted:* | *N/A* |

# Step 2: Classify the type of prediction model evaluation

Use the following table to classify the evaluation as model development, model validation or model update, or combination. Different signalling questions apply for different types of prediction model evaluation. If the evaluation does not fit one of these classifications then PROBAST should not be used.

| **Classify the evaluation based on its aim** | | |  |
| --- | --- | --- | --- |
| **Type of prediction study** | **PROBAST boxes to complete** | **Tick as appropriate** | **Definition for type of prediction model study** |
| Development only | Development | X | Prediction model development without external validation. These studies may include internal validation methods, such as bootstrapping and cross-validation techniques. |
| Development and validation | Development and validation |  | Prediction model development combined with external validation in other participants in the same article. |
| Validation only | Validation |  | External validation of existing (previously developed) model in other participants. |

| *This table should be completed once for each publication being assessed and for each relevant outcome in your review.* | |
| --- | --- |
| **Publication reference** | N/A |
| **Models of interest** | RF, XGBoost & LightGBM |
| **Outcome of interest** | Hypovigilance episode |

# Step 3: Assess risk of bias and applicability

PROBAST is structured as four key domains. Each domain is judged for risk of bias (low, high or unclear) and includes signalling questions to help make judgements. Signalling questions are rated as yes (Y), probably yes (PY), probably no (PN), no (N) or no information (NI). All signalling questions are phrased so that “yes” indicates absence of bias. Any signalling question rated as “no” or “probably no” flags the potential for bias; you will need to use your judgement to determine whether the domain should be rated as “high”, “low” or “unclear” risk of bias. The guidance document contains further instructions and examples on rating signalling questions and risk of bias for each domain.

The first three domains are also rated for concerns regarding applicability (low/ high/ unclear) to your review question defined above.

*Complete all domains separately for each evaluation of a distinct model. Shaded boxes indicate where signalling questions do not apply and should not be answered.*

| **DOMAIN 1: Participants** | | | |
| --- | --- | --- | --- |
| **A. Risk of Bias** | | | |
| *Describe the sources of data and criteria for participant selection:*  Electronic Health Record (HER)  Gateway (the patient’s bedside) | | | |
|  | | Dev | Val |
| 1.1 Were appropriate data sources used, e.g. cohort, RCT or nested case-control study data? | | Y |  |
| 1.2 Were all inclusions and exclusions of participants appropriate? | | Y |  |
| **Risk of bias introduced by selection of participants** | **RISK:**  *(low/ high/ unclear)* | **Low** |  |
| *Rationale of bias rating:* Patients were selected in one single ICU unit in Lévis, Québec containing medical and surgical ICU patients. Patients were screened daily and consecutive patients were considered for inclusion. | | | |
| **B. Applicability** | | | |
| *Describe included participants, setting and dates:* | | | |
| **Concern that the included participants and setting do not match CONCERN:**  **the review question** *(low/ high/ unclear)* | | | |
| *Rationale of applicability rating:* | | | |

| **DOMAIN 2: Predictors** | | |  |
| --- | --- | --- | --- |
| **A. Risk of Bias** | | |  |
| *List and describe predictors included in the final model, e.g. definition and timing of assessment:*  Heart rate (HR),Respiratory rate (RR), Oxygen saturation (SPO2-%),Pulse oximeter pulse rate (SPO2-R),noninvasive blood pressure (NBP-Diastolic,NBP-Systolic,NPB-Mean),Premature ventricular contraction (PVC), is the patient is intubated or not (CO2-EX_availability),if the patient has a rectal temperature sensor (temp_availability),if an arterial line is installed on the participant ( AR_availability) | | |  |
|  | | Dev | Val |
| 2.1 Were predictors defined and assessed in a similar way for all participants? | | Y |  |
| 2.2 Were predictor assessments made without knowledge of outcome data? | | Y |  |
| 2.3 Are all predictors available at the time the model is intended to be used? | | Y |  |
| **Risk of bias introduced by predictors or their assessment** | **RISK:**  *(low/ high/ unclear)* | **Low** |  |
| *Rationale of bias rating:*  All predictors were planned a priori for consideration in the models and all of the predictors are available at the time the model is intended to be used for identification. | | |  |
| **B. Applicability** | | |  |
| Concern that the definition, assessment or timing of predictors in the model do not match the review question | **CONCERN:**  *(low/ high/ unclear)* |  |  |
| *Rationale of applicability rating:* | | |  |

| **DOMAIN 3: Outcome** | | | |
| --- | --- | --- | --- |
| **A. Risk of Bias** | | | |
| *Describe the outcome, how it was defined and determined, and the time interval between predictor assessment and outcome determination:*  **The outcome is an hypovigilance episode or not hypovigilant episode. Experienced ICU nurses determined using the Richmond Agitation and Sedation Scale (RASS) and Ramsay Sedation Scale (RSS). The assessment was made hourly or sometime more when the nurse thought it was necessary.** | | | |
|  | | Dev | Val |
| 3.1 Was the outcome determined appropriately? | | yes |  |
| 3.2 Was a pre-specified or standard outcome definition used? | | Yes |  |
| 3.3 Were predictors excluded from the outcome definition? | | Yes |  |
| 3.4 Was the outcome defined and determined in a similar way for all participants? | | Yes |  |
| 3.5 Was the outcome determined without knowledge of predictor information? | | Yes |  |
| 3.6 Was the time interval between predictor assessment and outcome determination appropriate? | | PY |  |
| **Risk of bias introduced by the outcome or its determination** | **RISK:**  *(low/ high/ unclear)* | **Unclear** |  |
| *Rationale of bias rating:*  The RASS/RAMSAY outcome scale has been validated in numerous publications, and a clear link between the two scales has been established. The outcome was assessed hourly for all participants by trained ICU staff, and none of the predictors were included in the outcome definition. The predictors were unknown when determining the  The time interval was hourly most of the time and sometimes a little bit more when needed (more than hourly). The outcome may fluctuate over time, making it difficult to track, but ICU staff made efforts to monitor patients in the most appropriate manner. | | | |
| **B. Applicability** | | | |
| *At what time point was the outcome determined:*        *If a composite outcome was used, describe the relative frequency/distribution of each contributing outcome:* | | | |
| **Concern that the outcome, its definition, timing or determination do not match the review question** | **CONCERN:**  *(low/ high/ unclear)* |  |  |
| *Rationale of applicability rating:* | | | |

| **DOMAIN 4: Analysis** | | | |
| --- | --- | --- | --- |
| **Risk of Bias** | | | |
| *Describe numbers of participants, number of candidate predictors, outcome events and events per candidate predictor:*  Refer to Table 1 for patients' characteristics. There are 1493 hypovigilance episodes and 764 non-hypovigilant episodes. Refer to Figure 5 for predictors in the model. | | | |
| *Describe how the model was developed (for example in regards to modeling technique (e.g., survival or logistic modeling), predictor selection, and risk group definition):*  Predictor selection is explained on page 7 and the literature behind these predictor is explained in the introduction. | | | |
| *Describe whether and how the model was validated, either internally (e.g., bootstrapping, cross validation, random split sample) or externally (e.g., temporal validation, geographical validation, different settings, different types of participants):*  No external validation but a 10k fold group cross-validation was used to train the models. | | | |
| *Describe the performance measures of the model, e.g. (re)calibration, discrimination (re)classification, net benefit, and whether they were adjusted for optimism:*  Random search for fine-tuning the models. Binary classification (hypovigilance or not). | | | |
| *Describe any participants who were excluded from the analysis:*  No one was excluded. | | | |
| *Describe missing data on predictors and outcomes as well as methods used for missing data:*  We used backward and forward filling on predictors (2 minutes backward and then 2 minutes forward) on an 11-minute time series. No missing data on the outcome. | | | |
|  | | Dev | Val |
| 4.1 Were there a reasonable number of participants with the outcome? | | PN |  |
| 4.2 Were continuous and categorical predictors handled appropriately? | | N |  |
| 4.3 Were all enrolled participants included in the analysis? | | Y |  |
| 4.4 Were participants with missing data handled appropriately? | | Y |  |
| 4.5 Was selection of predictors based on univariable analysis avoided? | | N | 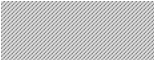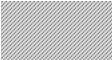 |
| 4.6 Were complexities in the data (e.g. censoring, competing risks, sampling of controls) accounted for appropriately? | | PY |  |
| 4.7 Were relevant model performance measures evaluated appropriately? | | Y |  |
| 4.8 Were model overfitting and optimism in model performance accounted for? | | Y | 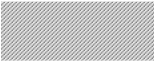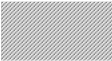 |
| 4.9 Do predictors and their assigned weights in the final model correspond to the results from multivariable analysis? | | NI (no final model) | 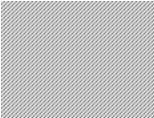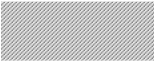 |
| **Risk of bias introduced by the analysis** | **RISK:**  *(low/ high/ unclear)* | **High** |  |
| *Rationale of bias rating:*  We have a small group of 30 participants, but a large number of episodes occurred. Having more participants would give us better insights. We had to simplify three variables, including two continuous ones (AR and CO2-ex), to deal with missing data; otherwise, we couldn't have used these variables at all. We didn't exclude any patients, even those who had zero hypovigilance episodes.  Explanation of back-forward filling: It's a method we used to handle missing data in our vital signs recordings. We filled in missing values by looking at the data before and after the missing points.  Question 4.5 highlights an unclear bias because we selected features based on a Mann-Whitney test, which is a univariate method.  We used cross-validation to prevent overfitting and properly evaluated the model across multiple metrics. | | | |

# Step 4: Overall assessment

| **Reaching an overall judgement about risk of bias of the prediction model evaluation** | |
| --- | --- |
| **Low risk of bias** | If all domains were rated low risk of bias.  If a prediction model was developed without any external validation, and it was rated as low risk of bias for all domains, consider downgrading to **high risk of bias**. Such a model can only be considered as low risk of bias, if the development was based on a very large data set and included some form of internal validation. |
| **High risk of bias** | If at least one domain is judged to be at **high risk of bias**. |
| **Unclear risk of bias** | If an unclear risk of bias was noted in at least one domain and it was low risk for all other domains. |

| **Reaching an overall judgement about applicability of the prediction model evaluation** | |
| --- | --- |
| **Low concerns regarding applicability** | If low concerns regarding applicability for all domains, the prediction model evaluation is judged to have **low concerns regarding applicability**. |
| **High concerns regarding applicability** | If high concerns regarding applicability for at least one domain, the prediction model evaluation is judged to have **high concerns regarding applicability**. |
| **Unclear concerns regarding applicability** | If unclear concerns (but no “high concern”) regarding applicability for at least one domain, the prediction model evaluation is judged to have **unclear concerns regarding applicability** overall. |

Use the following tables to reach overall judgements about risk of bias and concerns regarding applicability of the prediction model evaluation (development and/or validation) across all assessed domains. *Complete for each evaluation of a distinct model.*

| **Overall judgement about risk of bias and applicability of the prediction model evaluation** | | |
| --- | --- | --- |
| **Overall judgement of risk of bias** | **RISK:**  *(low/ high/ unclear)* | **High** |
| *Summary of sources of potential bias:*  Small dataset  No multivariable analysis before the ML models but univariate analysis was done for feature selection. | | |
| **Overall judgement of applicability** | **CONCERN:**  *(low/ high/ unclear)* | **-** |
| *Summary of applicability concerns:* | | |
