# Supplementary material for: Predicting Episodes of Hypovigilance in Intensive Care Units Using Routine Physiological Parameters and Artificial Intelligence: Derivation Study
Source: JMIR AI. 2025 Aug 27;4:e60885. doi: 10.2196/60885 (PMC12384691; doi:10.2196/60885)
Supplement: Multimedia Appendix 2 [file ai-v4-e60885-s002.docx]

The Glasgow Coma Scale was initially developed in 1974 at the University of Glasgow by professors of neurosurgery Bryan Jennett and Graham Teasdale. The scale enables the objective description of the level of impaired consciousness in patients experiencing acute illness or trauma. It employs three dimensions to evaluate patients: eye-opening, motor, and vocal responses. A succinct and intelligible representation of a patient's condition can be achieved by reporting each of these individually [1].

The Pfeffer Functional Activities Questionnaire (FAQ) is a tool for quickly identifying and assessing a loss of functional autonomy. It is used with participating patients or caregivers (to the best of their knowledge). It takes 5 to 10 minutes to complete. The questionnaire consists of 10 items that address the performance of daily and household activities such as meal preparation, outdoor mobility, financial management, etc. In the context of this project, the FAQ was administered by a member of the research team during the admission interview to identify if the participant presented any functional impairments, a predisposing factor to delirium. If questions were not answered by the caregiver, they were administered to participants when they recovered their capacity to consent. Otherwise, data recovered from reviewing the notes of various healthcare professionals (e.g., social workers) could be used to determine the answers to the FAQ questions. Finally, we did not exclude any participant even if data was missing about the FAQ items [2].

Clinical Frailty Score (CFS): The CFS is a clinically validated tool for assessing participants and fitness based on their clinical judgment. The score ranges from 1 (very fit) to 9 (terminally ill). The CFS was completed at the study’s inception and could be determined directly with participants or with any substitute decision-maker on their behalf. Given hospital visitation restrictions during the COVID-19 pandemic lockdowns, the questionnaire could be conducted over the phone with substitute decision-makers and caregivers [3].

1. Jain S, Iverson LM. Glasgow Coma Scale. StatPearls Treasure Island (FL): StatPearls Publishing; 2024. PMID:30020670

2. Pfeffer RI, Kurosaki TT, Harrah CHJ, Chance JM, Filos S. Measurement of functional activities in older adults in the community. J Gerontol United States; 1982 May;37(3):323–329. PMID:7069156

3. Church S, Rogers E, Rockwood K, Theou O. A scoping review of the Clinical Frailty Scale. BMC Geriatr 2020 Oct 7;20(1):393. PMID:33028215
